# A Novel Fault Detection with Minimizing the Noise-Signal Ratio Using Reinforcement Learning

**DOI:** 10.3390/s18093087

**Published:** 2018-09-13

**Authors:** Dapeng Zhang, Zhiling Lin, Zhiwei Gao

**Affiliations:** 1 School of Electrical and Information Engineering, Tianjin University, Tianjin 300072, China; zdp@tju.edu.cn; 2 School of Electrical Engineering, Tianjin University of Technology, Tianjin 300384, China; 3 Faculty of Engineering and Environment, University of Northumbria, Newcastle upon Tyne NE2 8ST, UK; zhiwei.gao@northumbria.ac.uk

**Keywords:** fault detection, reinforcement learning, noise-signal ratio

## Abstract

In this paper, a reinforcement learning approach is proposed to detect unexpected faults, where the noise-signal ratio of the data series is minimized to achieve robustness. Based on the information of fault free data series, fault detection is promptly implemented by comparing with the model forecast and real-time process. The fault severity degrees are also discussed by measuring the distance between the healthy parameters and faulty parameters. The effectiveness of the algorithm is demonstrated by an example of a DC-motor system.

## 1. Introduction

With the increasing expense and complexity of modern industrial systems, there is a growing demand for higher reliability and security. Measurement instrument faults may result in performance degradation or even malfunction due to the incorrect conclusion drawn by the process fault detection and diagnosis system. Therefore, the problem of fault detection and diagnosis (FDD) has become a popular research topic [1,2,3].

Generally, fault diagnosis methods can be categorized into model-based methods, signal-based methods and knowledge-based methods [1,2]. In model-based methods, the models of the industrial processes or the practical systems are obtained by using either physical principles or system identification techniques. Based on the model, fault diagnosis algorithms are developed to monitor the consistency between the measured outputs of the practical systems and the model-predicted outputs. Signal-based methods utilize measured signals rather than explicit input-output models for fault diagnosis. The feature signals to be extracted for symptom (or pattern) analysis can be either the time domain (e.g., mean, trends, standard deviation, phases, slope and magnitudes such as peak and root mean square) or frequency domain (e.g., spectrum). These issues were studied by various signal processing methods, such as wavelet transform (WT) [4], empirical mode decomposition (EMD) [5,6], intrinsic mode functions (IMF) [7] and local mean decomposition (LMD) [8]. A large volume of data has been more accessible with the development of modern electronic and measurement technologies such as SCADA and smart sensors [9,10,11,12,13], which stimulates knowledge-based fault diagnosis methods. Applying a variety of artificial intelligent techniques (either symbolic intelligence or computing intelligence) to the available historic data of the industrial processes, the underlying knowledge, which implicitly represents the dependence of the system variables, can be extracted. Interesting results on knowledge-based fault diagnosis and applications were reported during the last few decades [14,15,16,17,18].

Unexpected faults may cause performance degradation or even malfunction, and it is thus desired to detect, isolate and identify the faulty components as early as possible. However, it is difficult to release the fault feature in a short time because of the influences from heavy background noises. Based on the statistical theory, the traditional data-driven methods can be implemented by the sliding window technology in which the data are regarded as a concentration of system character and renew with window sliding. The features of the system can be extracted by analysing the data series in a sliding window after a filtering process and further stressed by strengthening technology such as PCA [19], SVM [20], information theory [21], and so forth. These traditional approaches have two flaws for fault detection: The first is that more data examples need to be collected in order to achieve a change of statistical character with a fault occurrence because a few new data can only have a small impact on the statistical character of the whole window. More data examples require more time to collect. Therefore, it is difficult for the traditional sliding window-based technology to carry out swift fault detection. The second is the lack of effective data in the case of early unexpected fault. Due to the complexity, uncertainty and unpredictability of the faults, it is challenging to obtain a number of valid fault data within a short period except for some special cases such as batch process. It is trade-off between getting more faulty data and giving less admissible time.

It is well known that the model parameters are more reliable than the state variables, especial in a noisy condition. However, the model parameters also face two problems similar to the aforementioned ones. The traditional approaches struggle to provide a quick detection due to the lack of the early information on sudden and unexpected faults.

Reinforcement learning (RL) is a powerful tool, which is motivated by statistics, psychology, neuroscience and computer science [22,23,24]. An agent will learn through experience, without a teacher. In each training session, named an episode, the agent explores the environment and receives the reward if any until it reaches the desired goal. The purpose of the training is to enhance the ‘brain’ of the agent. The goal of an agent is to maximize the reward that is received in the long run. One can obtain the optimal action only using the current states [25,26,27,28].

Motivated by the idea of “obtain the optimal action only using the current states”, an original idea based on RL is proposed to solve the swift fault detection problem. The minimization of the noise-signal ratio (NSR) is taken as the goal of the expecting series, and the policy iteration of RL is used as a tool to get parameters by considering the parameters as actions of RL. Then, one can get the model parameters corresponding to current states with noises. By comparing with the noise information (it is easier to get offline from the healthy data series), one will implement prompt fault detection and diagnosis with the next sample data. There are two main contributions in this paper.

(1) The unexpected faults will be detected promptly within a sampling period by using the measured data only.

(2) The estimated model is always consistent with the real-time process under the noisy condition by adjusting the parameters every sampling with the goal of minimizing the NSR using RL technology.

## 2. Problem Description and Preliminaries

### 2.1. Problem Description

**Figure 1:**
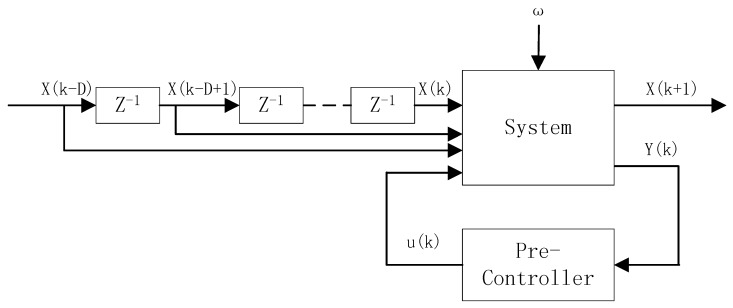
The structure of the system.

Suppose a discrete-time system with noises is controlled by a pre-controller, depicted by Figure 1.

Here, $x\left(k-D\right),x\left(k-D+1\right),x\left(k\right),\cdots ,x\left(k+1\right)\in R^{n}$ are the system states at sampling time k−D,k−D+1,k,⋯,k+1 respectively, and *D* is the order of the system. $u\left(k\right)\in R^{m},y\left(k\right)\in R^{p}$ are the control input and measured output, respectively; $\omega \left(k\right)\in R^{n}$ is a white Gaussian signal with zero mean and covariance matrix $\sum_{\omega }$. We suppose the system states are observable, and the control series $\left\{u\left(k\right)|u\left(k\right)\in R^{m},k=1,2,\cdots ,\right\}$ is obtained from the pre-controller’s output.

Let $\phi \left(k\right)={\left[x^{T}\left(k-D\right)x^{T}\left(k-D+1\right)\cdots x^{T}\left(k\right)u^{T}\left(k\right)\right]}^{T}\in R^{Dn+m}$; the system can be rewritten as a vector form:

(1) $$x_{m}\left(k+1\right)=\theta^{T}\phi \left(k\right)+\omega \left(k\right)$$

where $\theta^{T}=\left[\begin{array}{ccc} \theta_{1} & \cdots & \theta_{1,Dn+m}\\ \cdots & \ddots & \cdots \\ \theta_{n,1} & \cdots & \theta_{n,Dn+m} \end{array}\right]\in R^{n\times (Dn+m)}$ is a parameter matrix and *T* represents a transpose.

### 2.2. Noise-Signal Ratio

The noise is categorized into multiplicative noises and additive noise. Here, we only take into consideration additive noise, which is consistent with the nature of many processes. This means $x\left(k\right)=x^{*}\left(k\right)+\omega \left(k\right)$ for any time *k*, where x(k) is the observed system states, $x^{*}\left(k\right)$ is the real data without noise and ω(k) is the noise.

Define a noise-signal ratio $\delta_{i}$ of *i*-th-component of data series $\left\{x\left(k\right)|x\left(k\right)\in R^{n},k=1,2,\cdots ,l\right\}$ as:

(2) $$\delta_{i}=\frac{\sqrt{\sum_{k=1}^{l}{\left[x_{i}\left(k\right)-x_{i}^{*}\left(k\right)\right]}^{2}}}{\sqrt{\sum_{k=1}^{l}x_{i}^{*}{\left(k\right)}^{2}}}$$

where $x_{i}\left(k\right)$ and $x_{i}^{*}\left(k\right)$ are the *i*-th component of the measured data and the real data at *k* sampling time, respectively, and *l* is the length of the data series. Further, an integer noise-signal ratio δ of data series $\left\{x\left(k\right)|x\left(k\right)\in R^{n},k=1,2,\cdots ,l\right\}$ for an additive noise is:

(3) $$\delta =\sum_{i=1}^{n}\delta_{i}=\sum_{i=1}^{n}\frac{\sqrt{\sum_{k=1}^{l}{\left[x_{i}\left(k\right)-x_{i}^{*}\left(k\right)\right]}^{2}}}{\sqrt{\sum_{k=1}^{l}x_{i}^{*}{\left(k\right)}^{2}}}$$

There are three factors that affect the noise-signal ratio $\delta_{i}$ for a given *n*-dimensional data series: the measured data $\left\{x_{i}\left(k\right)\right\}$, the real data $x_{i}^{*}\left(k\right)$ and the length *l*. From the statistics viewpoint, *l* must have enough length in order to discover the feature of data series. This means it will spend a long time collecting the sample data. If one pursues a short time, the length *l* should be shorter. It is evident that when *l* becomes shorter, the noise will have a greater effect on the statistics character of the measured data series. It is a compromise between accuracy and velocity.

### 2.3. Reinforcement Learning Method

**Figure 2:**
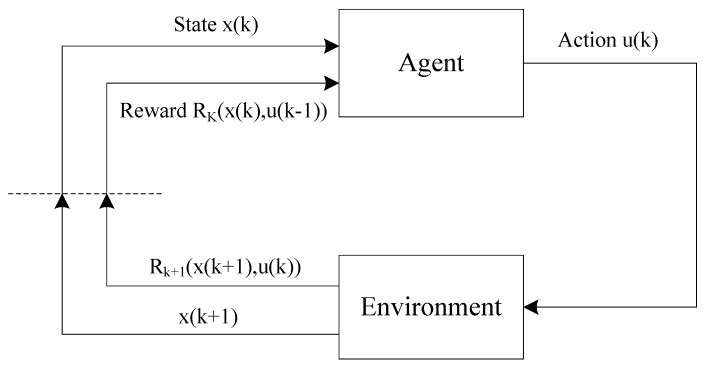
The basic frame of reinforcement learning.

The reinforcement learning that is motivated by statistics, psychology, neuroscience and computer science is a powerful tool to deal with uncertain surroundings by interacting with its environment. In terms of [22,24,25], the basic theory and methods of the reinforcement-learning are simply introduced here. The basic frame of reinforcement learning is shown in Figure 2 [24].

An agent will get the evaluation of good or bad behaviour on the environment and learn through experience without a teacher, who teaches how to do perform this. In every single training session, named an episode, the agent explores the environment by changing action $u_{i}$ and receives the state $x_{i}$ and the reward $R_{i}$. The purpose of the training is to enhance the ‘brain’ of the agent. The goal of an agent is to maximize the reward $\sum R_{i}$ that is received in the long run.

Consider a Markov decision process MDP(X,U,P,ℜ), where X is a set of states and U is a set of actions or controls. The transition probabilities P: X×U×X→[0,1] represent for each state x∈X and action u∈U the conditional probability P(x(k+1),x(k),u(k))=Pr{x(k+1)∣x(k),u(k)} of transitioning to state x(k+1)∈X where the MDP is in state x(k) and takes action u(k). The cost function ℜ:X×U×X→R is the expected immediate cost $R_{k}\left(x\left(k+1\right),x\left(k\right),u\left(k\right)\right)$ paid after transition to state x(k+1)∈X, given that the MDP starts from state x(k)∈X and takes action u(k)∈U. The value of a policy $V_{k}^{\pi }\left(x\left(k\right)\right)$ is defined as the conditional expected value of the future cost $E_{\pi }\left\{\sum_{i=k}^{k+T}\gamma^{i-k}R_{i}\right\}$, with $R_{i}\in R$ when starting in state x(k) at time *k* and following policy π(x,u). One can further have:

(4) $$\begin{array}{c} V_{k}^{\pi }\left(x\right)=E_{\pi }\left\{\sum_{i=k}^{k+T}\gamma^{i-k}R_{i}\right\}\\ =\sum_{u}\pi \left(x,u\right)\sum_{x(k+1)}P\left(x\left(k+1\right),x\left(k\right),u\left(k\right)\right)\left[R_{k}\left(x\left(k+1\right),x\left(k\right),u\left(k\right)\right)+\gamma E_{\pi }\left\{\sum_{i=k+1}^{k+T}\gamma^{i-(k+1)}R_{i}\right\}\right]\\ =\sum_{u}\pi \left(x,u\right)\sum_{x(k+1)}P\left(x\left(k+1\right),x\left(k\right),u\left(k\right)\right)\left[R_{k}\left(x\left(k+1\right),x\left(k\right),u\left(k\right)\right)+\gamma V_{k+1}^{\pi }\left(x\left(k+1\right)\right)\right] \end{array}$$

where T=∞. It is noted that T=∞ represents that the Markov decision process has enough length to show its essential characteristic according to the statistical law. If it is too short, the $V_{k}^{\pi }\left(x\right)$ is prone to inaccuracy with few data. We usually use enough length *l* instead of *∞* in practical application.

Equation (4) releases the value function $V_{k}^{\pi }\left(x\right)$ for the policy π(x,u) satisfying the Bellman Equation [29]:

(5) $$V_{k}^{\pi }\left(x\right)=\sum_{u}\pi \left(x,u\right)\sum_{x(k+1)}P\left(x\left(k+1\right),x\left(k\right),u\left(k\right)\right)\left[R_{k}\left(x\left(k+1\right),x\left(k\right),u\left(k\right)\right)+\gamma V_{k+1}^{\pi }\left(x\left(k+1\right)\right)\right]$$

Therefore, the optimal actions can be gained by alternating the policy evaluation and policy improvement according to Equations (6) and (7):

(6) $$V_{k}\left(x\right)=\sum_{u}\pi_{k}\left(x,u\right)\sum_{x(k+1)}P\left(x\left(k+1\right),x\left(k\right),u\left(k\right)\right)\left[R_{k}\left(x\left(k+1\right),x\left(k\right),u\left(k\right)\right)+\gamma V_{k}\left(x\left(k+1\right)\right)\right]$$

(7) $$\pi_{k}\left(x,u\right)=\underset{\pi }{\arg\min}\sum_{x(k+1)}P\left(x\left(k+1\right),x\left(k\right),u\left(k\right)\right)\left[R_{k}\left(x\left(k+1\right),x\left(k\right),u\left(k\right)\right)+\gamma V_{k}\left(x\left(k+1\right)\right)\right]$$

where γ is a discount factor with 0≤γ<1 in order to be convergent.

For a deterministic system, $\sum_{u}\pi_{k}\left(x,u\right)\sum_{x(k+1)}P\left(x\left(k+1\right),x\left(k\right),u\left(k\right)\right)=1$. As a result, Equations (6) and (7) are rewritten as:

(8) $$V_{k}\left(x\right)=R_{k}\left(x\left(k+1\right),x\left(k\right),u\left(k\right)\right)+\gamma V_{k}\left(x\left(k+1\right)\right)$$

(9) $$\pi_{k}\left(x,u\right)=\underset{\pi }{\arg\min}R_{k}\left(x\left(k+1\right),x\left(k\right),u\left(k\right)\right)+\gamma V_{k}\left(x\left(k+1\right)\right)$$

It is stressed that x(k+1) is only a temporary expected state in the process of alternating the policy evaluation and policy improvement, which is used to implement the cost $R_{k}\left(x\left(k+1\right),x\left(k\right),u\left(k\right)\right)$. The policy improvement (9) is usually obtained by using the greedy method [24] that will pursue the better policy at each iteration.

**Remark** **1.** *There is only state information in Equations (8) and (9). One can obtain the optimal action only using the two states x(k) and x(k+1) in the process of minimizing the goal $R_{k}$. It does not need more time to collect more data, and the past information is not necessary to know.*

## 3. Proposed Methodology

### 3.1. The System Reconfiguration and Parameter Acquisition

#### 3.1.1. Fault-Free Scenario

One can obtain the estimated Equation of System (1) as follows:

(10) $$\hat{x}\left(k+1\right)={\hat{\theta }}^{T}\phi \left(k\right)={\left[{\hat{\theta }}_{1}^{T}{\hat{\theta }}_{2}^{T}\cdots {\hat{\theta }}_{n}^{T}\right]}^{T}\phi \left(k\right)$$

where $\hat{x}\left(k+1\right)$ is an estimated value of x(k+1); ${\hat{\theta }}_{1},\cdots ,{\hat{\theta }}_{n}$ are vector components of $\hat{\theta }$. If there are enough data in data series with length *l*, the parameter $\hat{\theta }$ can be gained by using a least squares method (LSM) [30] according to the following:

(11) $$\hat{\theta }={\left[\phi^{T}\phi \right]}^{-1}\phi^{T}x_{k+1,l}$$

where $\phi ={\left[\phi_{1},\phi_{2},\cdots ,\phi_{k},\cdots ,\phi_{l}\right]}^{T},\phi_{k}={\left[x_{k-D,1},\cdots ,x_{k-D,n},\cdots ,x_{k,1},\cdots ,x_{k,n},u_{k,1},\cdots ,u_{k,m}\right]}^{T}\in R^{Dn+m}$, $x_{k+1,l}={\left[x_{k+1,1},\cdots ,x_{k+1,l}\right]}^{T}$ and the subscripts *k* and k+1 are the sampling time instants, while *l* is the length of the data series. The accuracy of $\hat{\theta }$ is further improved online by a recursion Equation (12) with new data $x_{k+1,l}$:

(12) $$\begin{array}{c} {\hat{\theta }}_{k+1}={\hat{\theta }}_{k}+\frac{P_{k}\phi \left[x_{k+1,l+1}-\phi^{T}\hat{\theta }\right]}{1+\phi^{T}P_{k}\phi }\\ P_{k+1}=P_{k}-\frac{P_{k}\phi \phi^{T}}{1+\phi^{T}P_{k}\phi }P_{k}\\ P_{k}=P_{0} \end{array}$$

where *P* is an auxiliary matrix and $P_{0}=\beta I$ for some large positive constant β; and ${\hat{\theta }}_{k+1}$ is an estimated parameter improved by adding new data.

Goodwin and Sin [30] showed that LSM converges asymptotically to the true parameters if $\hat{\theta }$ is fixed and ϕ(k) satisfies the persistent excitation condition:

(13) $$\epsilon_{0}I\leq \frac{1}{N}\sum_{k=1}^{N}\phi \left(k\right)\phi^{T}\left(k\right)\leq {\bar{\epsilon }}_{0}I$$

for all $N\geq N_{0}$, where $\epsilon_{0}\leq {\bar{\varepsilon }}_{0}$ and $N_{0}$ is a positive number. This indicates $x^{*}=\hat{x}$ in the meaning of the LSM. Here, $x^{*}$ is the real data without noise, and $\hat{x}$ is an estimated value by using LSM.

#### 3.1.2. Fault Scenario

It is assumed that the change from the normal to faulty operation does not affect the noise distribution and intensity. A model of data series subjected to a fault $\omega_{f}$ is described as:

(14) $$x_{f}\left(k+1\right)=\theta_{f}^{T}\phi_{f}\left(k\right)+\omega \left(k\right)+\omega_{f}\left(k\right)$$

where $\theta_{f}\in R^{Dn+m}$ is a coefficient vector after fault, ω(k) is the noise that is the same as fault free and $\omega_{f}$ is an unexpected fault. One can obtain ${\hat{\theta }}_{f}$ by applying the least squares method again if there are enough valid data. The estimated model subjected to faults is as Equation (15):

(15) $${\hat{x}}_{f}\left(k+1\right)={\hat{\theta }}_{f}^{T}\phi_{f}\left(k\right)={\left[{\hat{\theta }}_{f1}^{T}{\hat{\theta }}_{f2}^{T}\cdots {\hat{\theta }}_{fn}^{T}\right]}^{T}\phi \left(k\right)$$

Substitute (10) and (15) into (2), hence the noise-signal ratio of fault free $\delta_{i}$ and of fault $\delta_{f,i}$ is Equations (16) and (17):

(16) $$\delta_{i}=\frac{\sqrt{\sum_{k=1}^{l}{\left[x_{i}\left(k\right)-{\hat{\theta }}_{i}^{T}\phi \left(k-1\right)\right]}^{2}}}{\sqrt{\sum_{k=1}^{l}{\left[{\hat{\theta }}_{i}^{T}\phi \left(k-1\right)\right]}^{2}}}$$

(17) $$\delta_{f,i}=\frac{\sqrt{\sum_{k=1}^{l}{\left[x_{fi}\left(k\right)-{\hat{\theta }}_{fi}^{T}\phi_{f}\left(k-1\right)\right]}^{2}}}{\sqrt{\sum_{k=1}^{l}{\left[{\hat{\theta }}_{fi}^{T}\phi_{fi}\left(k-1\right)\right]}^{2}}}$$

The integer noise-signal ratio of fault free δ and of fault $\delta_{f}$ is obtained by substituting (10) and (15) into (3):

(18) $$\delta =\sum_{i=1}^{n}\frac{\sqrt{\sum_{k=1}^{l}{\left[x_{i}\left(k\right)-{\hat{\theta }}_{i}^{T}\phi \left(k-1\right)\right]}^{2}}}{\sqrt{\sum_{k=1}^{l}{\left[{\hat{\theta }}_{i}^{T}\phi \left(k-1\right)\right]}^{2}}}$$

(19) $$\delta_{f}=\sum_{i=1}^{n}\frac{\sqrt{\sum_{k=1}^{l}{\left[x_{fi}\left(k\right)-{\hat{\theta }}_{fi}^{T}\phi_{f}\left(k-1\right)\right]}^{2}}}{\sqrt{\sum_{k=1}^{l}{\left[{\hat{\theta }}_{fi}^{T}\phi_{fi}\left(k-1\right)\right]}^{2}}}$$

**Remark** **2.** *The noise-signal ratio $\delta_{f,i}$ and $\delta_{f}$ subjected to fault has a similar form as the noise-signal ratio $\delta_{i}$ and δ that is fault free. One can get ${\hat{\theta }}_{i}$ by the LSM method because there are enough valid data that are fault free. However, it is impracticable for ${\hat{\theta }}_{fi}$ in the early fault due to lack of effective data subject to limited time.*

*The noise-signal ratio for a data series that is given a dimension n and a length l is related to three factors: the current measured data {x(k)}, parameter ${\hat{\theta }}_{i}$ and the historical inputs ϕ(k−1) in the condition of either fault or fault free. When l=1, Equation (19) becomes Equation (20):*

(20) $$\delta_{f}\left(k\right)=\sum_{i=1}^{n}\frac{\sqrt{{\left[x_{fi}\left(k\right)-{\hat{\theta }}_{fi}^{T}\phi_{f}\left(k-1\right)\right]}^{2}}}{\sqrt{{\left[{\hat{\theta }}_{fi}^{T}\phi_{fi}\left(k-1\right)\right]}^{2}}}$$

*The noise-signal ratio $\delta_{f}\left(k\right)$ of single sample $x_{fi}\left(k\right)$ is referred to by using the input $\phi_{f}\left(k-1\right)$ and responding parameter ${\hat{\theta }}_{fi}^{T}$ at sample k. The other way around, one can get ${\hat{\theta }}_{fi}^{T}$ at sample k by using $\delta_{f}\left(k\right)$ in the case of knowing $x_{fi}\left(k\right)$ and $\phi_{f}\left(k-1\right)$.*

### 3.2. The Relation between Noise-Signal Ratio and Parameter

**Theorem** **1.** *For a data series {x(k),k=0,⋯,l}, the following conclusions are obtained if it is written as the form of Equation (1):*

*1. Different $\omega_{f}$ induce different ${\hat{\theta }}_{f}$;*

*2. The same ${\hat{\theta }}_{f}$ causes the same noise-signal ratio $\delta_{f}$;*

*3. Different ${\hat{\theta }}_{f}$ incurs different noise-signal ratio $\delta_{f,i}$.*

**Proof.**  1. For a measured data series {x(0),x(1),⋯,x(k),⋯,x(l)} subjected to fault and noise, it can be described by:

(21) $$x\left(k+1\right)={\hat{\theta }}_{f}^{T}\phi \left(k\right)+\omega \left(k\right)+\omega_{f}\left(k\right)$$

where ${\hat{\theta }}_{f}$ is the parameter by LSM and $\phi \left(k\right)={\left[x^{T}\left(k-D\right),x^{T}\left(k-D+1\right)\cdots x^{T}\left(k\right)\right]}^{T}$.

For a fault denoted by $\omega_{f1}\left(k\right)$, the data series can be written as:

(22) $$x_{f1}\left(k\right)={\hat{\theta }}_{f1}^{T}\phi_{f1}\left(k-1\right)+\omega \left(k\right)+\omega_{f1}\left(k\right)$$

For a fault denoted by $\omega_{f2}\left(k\right)$, we are not sure whether the fault will change the parameter ${\hat{\theta }}_{f}$. Therefore, the data series can be written as:

(23) $$x_{f2}\left(k\right)={\hat{\theta }}_{f2}^{T}\phi_{f2}\left(k-1\right)+\omega \left(k\right)+\omega_{f2}\left(k\right)$$

where the subscripts $f_{1}$ and $f_{2}$ are used to distinguish the data and parameters under different faults.

It is noted that we discuss the data properties of a measured data series. As a result, $x_{f1}\left(k\right)=x_{f2}\left(k\right)$ and $\phi_{f1}\left(k-1\right)=\phi_{f2}\left(k-1\right)$.

We assume ${\hat{\theta }}_{f1}={\hat{\theta }}_{f2}$ when $\omega_{f1}\neq \omega_{f2}$. Therefore, we can have:

(24) $$0=x_{f1}\left(k\right)-x_{f2}\left(k\right)={\hat{\theta }}_{f1}^{T}\phi_{f1}\left(k-1\right)+\omega \left(k-1\right)+\omega_{f1}\left(k-1\right)-{\hat{\theta }}_{f2}^{T}\phi_{f2}\left(k-1\right)-\omega \left(k-1\right)-\omega_{f2}\left(k-1\right)$$

leading to $\omega_{f1}=\omega_{f2}$, which is contradiction. As a result, we can have ${\hat{\theta }}_{f1}\neq {\hat{\theta }}_{f2}$ when $\omega_{f1}\neq \omega_{f2}$.

2. According to the definition of Equation (2), we have:

(25) $$\frac{\delta_{f1,i}}{\delta_{f2,i}}=\frac{\sqrt{\frac{\sum_{k=1}^{l}{\left[x_{f1,i}\left(k\right)-{\hat{\theta }}_{f1i}^{T}\phi_{f1}\left(k-1\right)\right]}^{2}}{\sum_{k=1}^{l}{\left[{\hat{\theta }}_{f1}^{T}\phi_{f1}\left(k-1\right)\right]}^{2}}}}{\sqrt{\frac{\sum_{k=1}^{l}{\left[x_{f2,i}\left(k\right)-{\hat{\theta }}_{f2i}^{T}\phi_{f2}\left(k-1\right)\right]}^{2}}{\sum_{k=1}^{l}{\left[{\hat{\theta }}_{f2i}^{T}\phi_{f2}\left(k-1\right)\right]}^{2}}}}$$

For a measured data series {x(0),x(1),⋯,x(k),⋯,x(l)}, it is noted that $x_{f1}\left(k\right)=x_{f2}\left(k\right)$ and $\phi_{f1}\left(k-1\right)=\phi_{f2}\left(k-1\right)$. For ${\hat{\theta }}_{f1}={\hat{\theta }}_{f2}$, one thus has:

(26) $$\sqrt{\frac{\sum_{k=1}^{l}{\left[x_{f1,i}\left(k\right)-{\hat{\theta }}_{f1i}^{T}\phi_{f1}\left(k-1\right)\right]}^{2}}{\sum_{k=1}^{l}{\left[{\hat{\theta }}_{f1}^{T}\phi_{f1}\left(k-1\right)\right]}^{2}}}=\sqrt{\frac{\sum_{k=1}^{l}{\left[x_{f2,i}\left(k\right)-{\hat{\theta }}_{f2i}^{T}\phi_{f2}\left(k-1\right)\right]}^{2}}{\sum_{k=1}^{l}{\left[{\hat{\theta }}_{f2i}^{T}\phi_{f2}\left(k-1\right)\right]}^{2})}}$$

It is obvious that $\delta_{f1,i}\neq 0$ and $\delta_{f2,i}\neq 0$. Therefore, $\frac{\delta_{f1}}{\delta_{f2}}=1$. Therefore, $\delta_{f1}=\delta_{f2}$. This means the same ${\hat{\theta }}_{f}$ causes the same noise-signal ratio $\delta_{f,i}$. Further, it results in the same integer noise-signal ratio $\delta_{f}$ due to $\delta_{f}=\sum_{i=1}^{n}\delta_{f,i}$ according to Equation (3).

3. Arbitrary select the *i*-th component $x_{f,i}$ of $x_{f}$.

**Hypothesis** **1.**  *Different ${\hat{\theta }}_{f}$ have the same noise-signal ratio $\delta_{f}$, which means $\delta_{f1}=\delta_{f2}$. Observe that:*

(27) $$\sqrt{\frac{\sum_{k=1}^{l}{\left[x_{1}\left(k\right)-{\hat{\theta }}_{f1}^{T}\phi \left(k-1\right)\right]}^{2}}{\sum_{k=1}^{l}{\left[{\hat{\theta }}_{f1}^{T}\phi \left(k-1\right)\right]}^{2}}}=\sqrt{\frac{\sum_{k=1}^{l}{\left[x_{2}\left(k\right)-{\hat{\theta }}_{f2}^{T}\phi \left(k-1\right)\right]}^{2}}{\sum_{k=1}^{l}{\left[{\hat{\theta }}_{f2}^{T}\phi \left(k-1\right)\right]}^{2}}}$$

*which is equivalent to:*

(28) $$\frac{\sum_{k=1}^{l}{\left[x_{1}\left(k\right)-{\hat{\theta }}_{f1}^{T}\phi \left(k-1\right)\right]}^{2}}{\sum_{k=1}^{l}{\left[{\hat{\theta }}_{f1}^{T}\phi \left(k-1\right)\right]}^{2}}-\frac{\sum_{k=1}^{l}{\left[x_{2}\left(k\right)-{\hat{\theta }}_{f2}^{T}\phi \left(k-1\right)\right]}^{2}}{\sum_{k=1}^{l}{\left[{\hat{\theta }}_{f2}^{T}\phi \left(k-1\right)\right]}^{2}}=0$$

*Rearranging the Equation above, we have:*

(29) $$\sum_{k=1}^{l}{\left[{\hat{\theta }}_{f2}^{T}\phi \left(k-1\right)\right]}^{2}\sum_{k=1}^{l}{\left[x_{1}\left(k\right)-{\hat{\theta }}_{f1}^{T}\phi \left(k-1\right)\right]}^{2}-\sum_{k=1}^{l}{\left[{\hat{\theta }}_{f1}^{T}\phi \left(k-1\right)\right]}^{2}\sum_{k=1}^{l}{\left[x_{2}\left(k\right)-{\hat{\theta }}_{f2}^{T}\phi \left(k-1\right)\right]}^{2}=0$$

(30) $$\sum_{k=1}^{l}\sum_{j=1}^{l}{\left[{\hat{\theta }}_{f2}^{T}\phi \left(k-1\right)\right]}^{2}{\left[x_{1}\left(j\right)-{\hat{\theta }}_{f1}^{T}\phi \left(j-1\right)\right]}^{2}-\sum_{k=1}^{l}\sum_{j=1}^{l}{\left[{\hat{\theta }}_{f1}^{T}\phi \left(k-1\right)\right]}^{2}{\left[x_{2}\left(j\right)-{\hat{\theta }}_{f2}^{T}\phi \left(j-1\right)\right]}^{2}=0$$

(31) $$\sum_{k=1}^{l}\sum_{j=1}^{l}\left\{{\left[{\hat{\theta }}_{f2}^{T}\phi \left(k-1\right)\right]}^{2}{\left[x_{1}\left(j\right)-{\hat{\theta }}_{f1}^{T}\phi \left(j-1\right)\right]}^{2}-{\left[{\hat{\theta }}_{f1}^{T}\phi \left(k-1\right)\right]}^{2}{\left[x_{2}\left(j\right)-{\hat{\theta }}_{f2}^{T}\phi \left(j-1\right)\right]}^{2}\right\}=0$$

*Denote:*

(32) $$\begin{array}{c} \hbar ={\left[{\hat{\theta }}_{f2}^{T}\phi \left(k-1\right)\right]}^{2}{\left[x_{1}\left(j\right)-{\hat{\theta }}_{f1}^{T}\phi \left(j-1\right)\right]}^{2}-{\left[{\hat{\theta }}_{f1}^{T}\phi \left(k-1\right)\right]}^{2}{\left[x_{2}\left(j\right)-{\hat{\theta }}_{f2}^{T}\phi \left(j-1\right)\right]}^{2}\\ =\underset{\hbar 1}{\underset{︸}{{\left\{{\hat{\theta }}_{f2}^{T}\phi \left(k-1\right)\left[x_{1}\left(j\right)-{\hat{\theta }}_{f1}^{T}\phi \left(j-1\right)\right]-{\hat{\theta }}_{f1}^{T}\phi \left(k-1\right)\left[x_{2}\left(j\right)-{\hat{\theta }}_{f2}^{T}\phi \left(j-1\right)\right]\right\}}^{2}}}\\ +\underset{\hbar 2}{\underset{︸}{2{\hat{\theta }}_{f2}^{T}\phi \left(k-1\right)\left[x_{1}\left(j\right)-{\hat{\theta }}_{f1}^{T}\phi \left(j-1\right)\right]{\hat{\theta }}_{f1}^{T}\phi \left(k-1\right)\left[x_{2}\left(j\right)-{\hat{\theta }}_{f2}^{T}\phi \left(j-1\right)\right]-2{\left[{\hat{\theta }}_{f1}^{T}\phi \left(k-1\right)\right]}^{2}{\left[x_{2}\left(j\right)-{\hat{\theta }}_{f2}^{T}\phi \left(j-1\right)\right]}^{2}}} \end{array}$$

*by the matching squares method.*

*Let:*

(33) $$\hbar 1={{\hat{\theta }}_{f2}^{T}\phi \left(k-1\right)\left[x_{1}\left(j\right)-{\hat{\theta }}_{f1}^{T}\phi \left(j-1\right)\right]-{\hat{\theta }}_{f1}^{T}\phi \left(k-1\right)\left[x_{2}\left(j\right)-{\hat{\theta }}_{f2}^{T}\phi \left(j-1\right)\right]}^{2}\geq 0$$

(34) $$\begin{array}{c} \hbar 2=2{\hat{\theta }}_{f2}^{T}\phi \left(k-1\right)\left[x_{1}\left(j\right)-{\hat{\theta }}_{f1}^{T}\phi \left(j-1\right)\right]{\hat{\theta }}_{f1}^{T}\phi \left(k-1\right)\left[x_{2}\left(j\right)-{\hat{\theta }}_{f2}^{T}\phi \left(j-1\right)\right]-2{\left[{\hat{\theta }}_{f1}^{T}\phi \left(k-1\right)\right]}^{2}{\left[x_{2}\left(j\right)-{\hat{\theta }}_{f2}^{T}\phi \left(j-1\right)\right]}^{2}\\ =2\left\{\underset{\hbar 4}{\underset{︸}{{\hat{\theta }}_{f2}^{T}\phi \left(k-1\right)\left[x_{1}\left(j\right)-{\hat{\theta }}_{f1}^{T}\phi \left(j-1\right)\right]-{\hat{\theta }}_{f1}^{T}\phi \left(k-1\right)\left[x_{2}\left(j\right)-{\hat{\theta }}_{f2}^{T}\phi \left(j-1\right)\right]}}\right\}\underset{\hbar 3}{\underset{︸}{{\hat{\theta }}_{f1}^{T}\phi \left(k-1\right)\left[x_{2}\left(j\right)-{\hat{\theta }}_{f2}^{T}\phi \left(j-1\right)\right]}} \end{array}$$

*Further, let:*

(35) $$\hbar 3=\underset{x_{1}\left(k\right)}{\underset{︸}{\left({\hat{\theta }}_{f1}^{T}\phi \left(k-1\right)\right)}}\underset{\omega (j-1)}{\underset{︸}{\left[x_{2}\left(j\right)-{\hat{\theta }}_{f2}^{T}\phi \left(j-1\right)\right]}}$$

*Note ℏ3≠0 besides $x_{1}\left(k\right)=0$ or ω(j−1)=0 (scarcely):*

(36) $$\hbar 4={\hat{\theta }}_{f2}^{T}\phi \left(k-1\right)\left[x_{1}\left(j\right)-{\hat{\theta }}_{f1}^{T}\phi \left(j-1\right)\right]-\left[{\hat{\theta }}_{f1}^{T}\phi \left(k-1\right)\right]\left[x_{2}\left(j\right)-{\hat{\theta }}_{f2}^{T}\phi \left(j-1\right)\right]=\left[{\hat{\theta }}_{f2}^{T}-{\hat{\theta }}_{f1}^{T}\right]\phi \left(k-1\right)x_{1}\left(j\right)$$

*$\left(x_{1}\left(j\right)=x_{2}\left(j\right)\right)$ for the same series data.*

*If ℏ=0, there is ℏ1=0 and ℏ4=0. Further:*

(37) $$\left[{\hat{\theta }}_{f2}^{T}-{\hat{\theta }}_{f1}^{T}\right]\phi \left(k-1\right)=0$$

*Notice ${\hat{\theta }}_{f2}^{T}$ and ${\hat{\theta }}_{f1}^{T}$ are fixed by LSM and ϕ(k−1) is a vector from measured data, but it is uncertain for all k. There is no other vector to satisfy this Equation except ${\hat{\theta }}_{f2}^{T}-{\hat{\theta }}_{f1}^{T}=0$. Therefore, ${\hat{\theta }}_{f1}={\hat{\theta }}_{f2}$, which is contrary to the hypothesis.*

☐

**Remark** **3.** *The above analyses release the relationship between parameters ${\hat{\theta }}_{f}$ and noise-signal ratio $\delta_{f,i}$. One can eliminate the influence of noise by the most extent by adjusting the parameter ${\hat{\theta }}_{f}$ with the target of minimizing the NSR of data series. Once the parameter ${\hat{\theta }}_{f}$ is determined, the model is used to forecast the next state ${\hat{x}}_{k+1}$ without noise. Therefore, the measured state $x_{k+1}$ will be judged immediately according to the noise law based on the model prediction ${\hat{x}}_{k+1}$.*

The parameters ${\hat{\theta }}_{f}$ can be estimated by traditional methods such as LSM and MLE (maximum likelihood method) based on the historical numerical data. Window technology is used to reduce computational load, and the sliding window is employed to capture the time-varying parameters in the dynamic system. The statistics characteristics depend on the data in the window. A longer window, which includes more data, means higher accuracy, but needs more time to make a decision. A shorter window, which consists of less data, means a quick decision, but it also needs enough data in order to satisfy the statistics law.

### 3.3. Seeking ${\hat{\theta }}_{f}$ by the Reinforcement Learning Method

Engineering systems are subjected to faults or malfunctions due to unexpected events, which would degrade the operation performance and even lead to the operation failure. As a result, the fault should be detected quickly, and measures will be taken as early as possible. The greatest difficulty is the lack of enough valid data for an early fault. Reinforcement learning provides a way to estimate the parameters directly by approaching the noise-signal ratio $\delta_{f}$ of the fault to noise-signal ratio $\delta_{h}$ of health (fault free).

To apply the reinforcement learning, the first thing is to determine the cost function $R_{k}\left(\delta_{f}\left(k\right)\right)$ at time k. Here, one defines the cost function $R_{k}\left(\delta_{f}\left(k\right)\right)$ at time k as an absolute value of error between the current integer noise-signal ratio $\delta_{f}\left(k\right)$ and the integer noise-signal ratio $\delta_{h}$ of being fault free.

(38) $$R_{k}\left(\delta_{f}\left(k\right)\right)=|\delta_{f}\left(k\right)-\delta_{h}|=\sum_{i=1}^{n}\frac{\sqrt{{\left[x_{fi}\left(k\right)-{\hat{\theta }}_{fi}^{T}\phi_{f}\left(k-1\right)\right]}^{2}}}{\sqrt{{\left[{\hat{\theta }}_{fi}^{T}\phi_{fi}\left(k-1\right)\right]}^{2}}}-\delta_{h}$$

where $\delta_{h}$ is the integer noise-signal ratio of being fault free that will be achieved offline according to Equation (18), |·| is the absolute value and the meanings of other parameters are the same as before. The function $V_{k}\left(\delta_{f}\left(k\right)\right)$ after time k is defined as:

(39) $$V_{k}\left(\delta_{f}\left(k\right)\right)=\sum_{i=k}^{\infty }\gamma^{i-k}R_{i}\left(\delta_{f}\left(i\right)\right)$$

As a result, one has:

(40) $$V_{k}\left(\delta_{f}\left(k\right)\right)=R_{k}\left(\delta_{f}\left(k\right)\right)+\gamma V_{k+1}\left(\delta_{f}\left(k+1\right)\right)$$

Following a Bellman optimal principle, the optimal value function is obtained according to Equation (41):

(41) $$V^{*}\left(\delta_{f}\left(k\right)\right)=\min_{{\hat{\theta }}_{f}\left(k\right)}R_{k}\left(\delta_{f}\left(k\right)\right)+\gamma V_{k+1}\left(\delta_{f}\left(k+1\right)\right)$$

where $V^{*}\left(\delta_{f}\left(k\right)\right)$ and ${\hat{\theta }}_{f}\left(k\right)$ are the optimal value function and the parameter at time k, respectively; and γ is a discount factor, 0≤γ<1.

It is noticed that (41) cannot be used online because one cannot know the information of the future time instant, that is $\delta_{f}\left(k+1\right)$. A Q-algorithm proposed by Watkins [23] provides an effective solution by substituting the Q-function. A mimic of the Q-algorithm defines the evaluation function $Q(\delta_{f}\left(k\right),{\hat{\theta }}_{f}\left(k\right))$ as the minimum discounted cumulative reward that can be achieved from $\delta_{f}\left(k\right)$ and ${\hat{\theta }}_{f}\left(k\right)$ as the first action:

(42) $$Q\left(\delta_{f}\left(k\right),{\hat{\theta }}_{f}\left(k\right)\right)\overset{def}{=}R_{k}\left(\delta_{f}\left(k\right),{\hat{\theta }}_{f}\left(k\right)\right)+V^{*}\left(\varphi \left(\delta_{f}\left(k\right),{\hat{\theta }}_{f}\left(k\right)\right)\right)$$

where $\varphi (\delta_{f}\left(k\right),{\hat{\theta }}_{f}\left(k\right))$ expresses the state $\delta_{f}\left(k+1\right)$ that comes from $\delta_{f}\left(k\right)$ and ${\hat{\theta }}_{f}\left(k\right)$, that is $\delta_{f}\left(k+1\right)=\varphi (\delta_{f}\left(k\right)$, ${\hat{\theta }}_{f}\left(k\right))$. One denotes $\varphi (\delta_{f}\left(k\right),{\hat{\theta }}_{f}\left(k\right))$ in order to stress the relation between $\delta_{f}\left(k+1\right)$ and $\delta_{f}\left(k\right),{\hat{\theta }}_{f}\left(k\right)$. If Q achieves its optimization under some parameter ${\hat{\theta }}_{f}\left(k\right)$, the function V can also achieve its optimization with the same parameter. As a result, V may be replaced by Q. This implies that the optimal parameter can be obtained only by reward without using the value function V.

Denote the optimum of Q as $Q^{*}$; therefore, one has:

(43) $$\begin{array}{c} Q^{*}\left(\delta_{f}\left(k\right),{\hat{\theta }}_{f}\left(k\right)\right)=\min_{{\hat{\theta }}_{f}\left(k\right)}\left[R_{k}\left(\delta_{f}\left(k\right),{\hat{\theta }}_{f}\left(k\right)\right)+V^{*}\left(\varphi \left(\delta_{f}\left(k\right),{\hat{\theta }}_{f}\left(k\right)\right)\right)\right]\\ =R^{*}\left(\delta_{f}\left(k\right),{\hat{\theta }}_{f}\left(k\right)\right)+V^{*}\left(\delta_{f}\left(k+1\right)\right)=V^{*}\left(\delta_{f}\left(k\right),{\hat{\theta }}_{f}\left(k\right)\right) \end{array}$$

where the superscript * expresses the optimal values. It is seen from Equation (43) that $Q^{*}\left(\delta_{f}\left(k\right),{\hat{\theta }}_{f}\left(k\right)\right)$ is equivalent to $V^{*}\left(\delta_{f}\left(k\right),{\hat{\theta }}_{f}\left(k\right)\right)$ with the same parameter. Therefore, the optimal parameter ${\hat{\theta }}_{f}\left(k\right)$ can be obtained by the policy iteration that includes the alternation of two processes: policy evaluation and policy improvement following Equations (44) and (45):

(44) $$Q\left(\delta_{f}\left(k\right),{\hat{\theta }}_{f}\left(k\right)\right)=R\left(\delta_{f}\left(k\right),{\hat{\theta }}_{f}\left(k\right)\right)+\gamma \min_{{\hat{\theta }}_{f}\left(k+1\right)}Q\left(\delta_{f}\left(k+1\right),{\hat{\theta }}_{f}\left(k+1\right)\right)$$

(45) $$\pi_{k}\left(\delta_{f}\left(k\right),{\hat{\theta }}_{f}\left(k\right)\right)=\underset{{\hat{\theta }}_{f}\left(k+1\right)}{\arg\min}Q\left(\delta_{f}\left(k\right),{\hat{\theta }}_{f}\left(k\right)\right)$$

where $\pi_{k}$ is called a policy in reinforcement learning. By using policy iteration, it will finally converge to the steady state, and we get the responding parameter.

It is important for policy iteration to be convergent. Fortunately, it has been proven by Lemma 1.

**Lemma** **1** **([21]).** *Consider a Q learning agent in a deterministic Markov decision process (MDP) with bounded reward $\left(\forall \delta_{f}\left(k\right),{\hat{\theta }}_{f}\left(k\right)\right)\left|R_{k}\left(\delta_{f}\left(k\right),{\hat{\theta }}_{f}\left(k\right)\right)\right|\leq c$. The Q learning agent uses the training rule of Equation:*

$$Q_{k}\left(\delta_{f}\left(k\right),{\hat{\theta }}_{f}\left(k\right)\right)\leftarrow R_{k}\left(\delta_{f}\left(k\right),{\hat{\theta }}_{f}\left(k\right)\right)+\gamma \min_{{\hat{\theta }}_{f}\left(k+1\right)}Q_{k+1}\left(\delta_{f}\left(k+1\right),{\hat{\theta }}_{f}\left(k+1\right)\right)$$

*initializes its $Q_{k}\left(\delta_{f}\left(k\right),{\hat{\theta }}_{f}\left(k\right)\right)$ to arbitrary finite values and uses a discount factor γ such that 0≤γ<1. Let $Q_{k}^{\left(n\right)}\left(\delta_{f}\left(k\right),{\hat{\theta }}_{f}\left(k\right)\right)$ denote the agent’s hypothesis $Q_{k}\left(\delta_{f}\left(k\right),{\hat{\theta }}_{f}\left(k\right)\right)$ following the n-th update. If each state-action pair is visited infinitely often, then $Q_{k}^{\left(n\right)}\left(\delta_{f}\left(k\right),{\hat{\theta }}_{f}\left(k\right)\right)$ converges to $Q_{k}\left(\delta_{f}\left(k\right),{\hat{\theta }}_{f}\left(k\right)\right)$ as n→∞, for all $\delta_{f}\left(k\right),{\hat{\theta }}_{f}\left(k\right)$.*

**Remark** **4.** *Lemma 1 provides a guarantee on the convergence of Q learning. By using policy iteration, the Q learning agent will finally converge to the steady state, and the optimal control $\pi^{*}\left(\delta_{f}\left(k\right),{\hat{\theta }}_{f}\left(k\right)\right)$ can be obtained readily.*

Procedure 1:

The RL algorithm can be summarized as follows:

Step 1: Initialize $\hat{Q}\left(\delta_{f}\left(k\right),{\hat{\theta }}_{f}\left(k\right)\right)$ to zero.

Step 2: Select a parameter ${\hat{\theta }}_{f}\left(k\right)$ randomly.

Step 3: Receive immediate reward $R(\delta_{f}\left(k\right),{\hat{\theta }}_{f}\left(k\right))$ according to Equation (38).

Step 4: Get the new state $\delta_{f}\left(k+1\right)$ and compute the value function according to Equation (40).

Step 5: Update the $\hat{Q}\left(\delta_{f}\left(k+1\right),{\hat{\theta }}_{f}\left(k+1\right)\right)$ based on current state $\delta_{f}\left(k\right)$ according to Equation (41).

Step 6: Set the next state $\delta_{f}\left(k+1\right)$ as the current state $\delta_{f}\left(k\right)$.

Step 7: Repeat Steps 3–6 until it is convergent.

Step 8: Find the best parameter ${\hat{\theta }}_{f}^{*}\left(k\right)$ according to Equation (46).

### 3.4. Detection of Fault

Based on the parameters ${\hat{\theta }}_{f}^{*}\left(k\right)$, we will get the next state ${\hat{x}}_{f}\left(k+1\right)$ according to Equation (15). Therefore, we have a chance to judge new measure data $x_{f}\left(k+1\right)$ immediately with taking ${\hat{x}}_{f}\left(k+1\right)$ as a criterion.

The state ${\hat{x}}_{f}\left(k+1\right)$ with fault is made up of three parts: the real state $x^{*}\left(k+1\right)$ that is fault free, the component from fault $\omega_{f}$ and the component from noise ω. We take the first two items as an integer and remark that they are the real data $x_{f}^{*}\left(k+1\right)$ of $x_{f}\left(k+1\right)$. Considering the parameter ${\hat{\theta }}_{f}^{*}$ is obtained by seeking for a goal of minimizing the noise-signal ratio, Equation (15) implies the noise minimization of forecasting the state at the next time k+1. Therefore, $x_{f}^{*}\left(k+1\right)$ is obtained by ${\hat{\theta }}_{f}^{*}$ according to Equation (15). We will get the estimated state ${\hat{x}}_{f}\left(k+1\right)$ at time k+1 in the case of fault according to:

(46) $${\hat{x}}_{f}\left(k+1\right)={\hat{x}}_{f}^{*}\left(k+1\right)+e={\left({\hat{\theta }}_{f}^{*}\right)}^{T}\left(k\right)\phi_{f}\left(k\right)+e$$

where ${\hat{\theta }}_{f}^{*}\left(k\right)$ is the parameters at time k obtained from the RL algorithm, T is the transpose and $e={\left[e_{1},e_{2},\cdots ,e_{n}\right]}^{T}$ is the confidence interval of noise ω at confidence level α:

(47) $$e_{i}=\pm \sqrt{\frac{D_{i}}{l}}Z_{\alpha /2}$$

where $D_{i}$ is the variance of the i-th component of samples, which are obtained offline by data series $\left\{x\left(k\right)|x\left(k\right)\in R^{n},k=1,2,\cdots ,l\right\}$ that is fault free:

(48) $$D_{i}=\frac{1}{l-1}\sum_{j=1}^{l}{\left[x_{i}\left(j\right)-{\hat{x}}_{i}\left(j\right)\right]}^{2}$$

$Z_{\alpha /2}$ is a normal distribution.

The above analysis shows that one can forecast ${\hat{x}}_{f}\left(k+1\right)$ in a noisy condition only by using ϕ(k) during one sampling period. It is valuable for the system to detect faults promptly.

Define the Euclidean distance (ED) between measure $x_{f}\left(k+1\right)$ and estimation ${\hat{x}}_{f}\left(k+1\right)$ as:

(49) $$ED\left(k+1\right)=\sum_{i=1}^{n}{\left[x_{fi}\left(k+1\right)-{\hat{x}}_{fi}\left(k+1\right)\right]}^{2}$$

where $x_{f}\left(k+1\right)\in R^{n}$ and ${\hat{x}}_{f}\left(k+1\right)\in R^{n}$ are the measured data and the estimated data at time k+1 under the fault, respectively.

The threshold of ED is selected as the maximum error between measured data and estimated data being fault free:

(50) $$ED_{sh}=\max_{k}\left\{\sum_{i=1}^{n}{\left[x_{i}\left(k\right)-{\hat{x}}_{i}\left(k\right)\right]}^{2},k=1,\cdots ,l\right\}$$

One can detect a fault if:

(51) $$ED\left(k+1\right)>ED_{sh}$$

Once one detects a fault, the parameters that are fault free will keep unchanged in order to build a virtual healthy model. Meanwhile, the parameters subject to fault continue to renew by the proposed RL method and forecast the next state under fault. In this condition, the ED becomes an indicator of fault degree (IFD). Therefore, we get Equation (52) by replacing ${\hat{x}}_{f}\left(k+1\right)$ for fault with $\hat{x}\left(k+1\right)$ for fault free in Equation (49):

(52) $$IFD\left(k+1\right)=\sum_{i=1}^{n}{\left[{\hat{x}}_{fi}\left(k+1\right)-\hat{x}\left(k+1\right)\right]}^{2}$$

Here, ${\hat{x}}_{fi}\left(k+1\right)$ for minimizing NSR is used to instead of $x_{fi}\left(k+1\right)$ in order to reduce the effect of noise. We use the IFD(k+1) to express the severity of the fault at k+1, so we will evaluate the fault degree in time and take measures to balance the safety and efficiency of the plant.

**Remark** **5.** *One will detect a fault and evaluate the fault degree promptly during one sampling period according to Equations (51) and (52).*

*The forecast of states at k+1 is valid under faulty or under fault-free conditions because the parameters of a reference model are essentially obtained by minimizing the noise-signal ratio.*

*This method only makes use of the residual and noise-signal ratio so that it is easy to identify the condition under being fault free. Meanwhile, it has the ability to trace unexpected fault by adjusting the parameters online.*

Procedure 2:

The fault detection and fault seriousness degree procedure is given as follows:

Step 1. Get the next real state ${\hat{x}}_{f}^{*}\left(k+1\right)$ without the noise based on the parameters ${\hat{\theta }}_{f}^{*}\left(k\right)$ from Procedure 1 according to Equation (15).

Step 2. Computer the variance of the i-th component of samples from the data series being fault free according to Equation (48).

Step 3. Get the estimated state ${\hat{x}}_{f}\left(k+1\right)$ according to Equation (7).

Sept 4. Get the measured data $x_{f}\left(k+1\right)$.

Step 5. Compute the Euclidean distance (ED) between measure $x_{f}\left(k+1\right)$ and estimation ${\hat{x}}_{f}\left(k+1\right)$ according to Equation (49).

Step 6. Compute the threshold of ED according to Equation (50).

Step 7. Perform fault detection and get the fault seriousness degree according to (51) and (52).

Step 8. Go to Step 1 to check the next state.

## 4. Examples and Simulations

**Figure 3:**
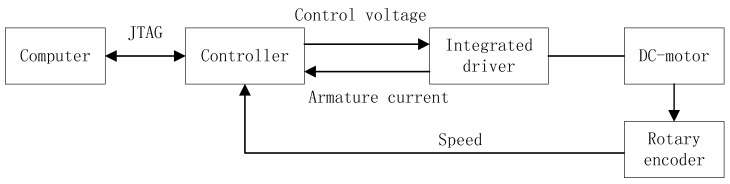
The topology of DC-motor test bed.

In this section, simulation results based on a DC-motor are presented to verify the efficacy of the proposed scheme. Figure 3 shows the topology of the DC-motor test bed.

**Figure 4:**
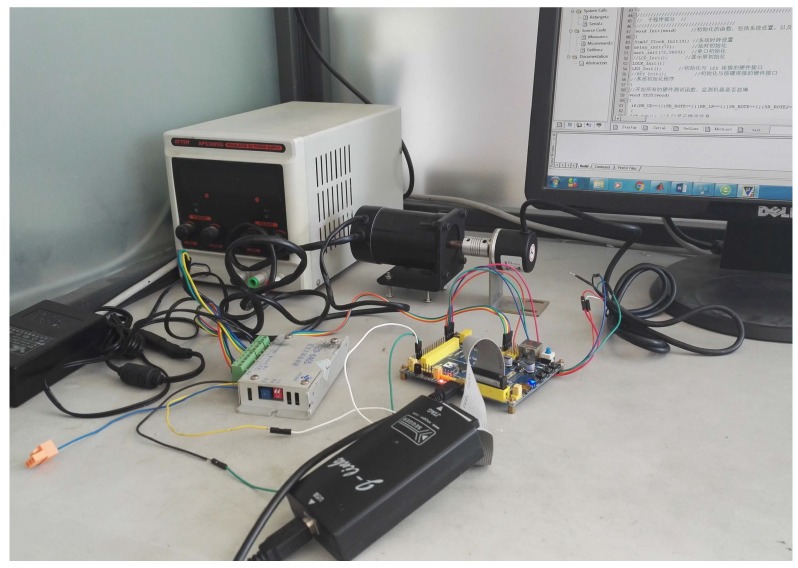
The test bed of the DC-motor.

The DC-motor is selected as Model 57BL90-210 with 24 V, 1000 rpm and 60 W. The rotary encoder is LPD3806-600BM. The integrated driver is an improved ZD-6405 that provides the positive inversion with a toggle switch and speed governing with 0–5V control voltage. It also gives the armature current detection and some protections against short circuit, under voltage and overload. The DC-motor is driven by an integrated driver with the controller of the STM32 single-chip microcomputer. The controller of STM32 is used to receive the DC-motor speed collected by the rotary encoder and the armature current from the integrated driver and, meanwhile, to output the driver control voltage according to the control approach. The controller is programmed on the plat of Keil3.0 by the JTAG (Joint-Test-Action-Group) interface, and the data are transmitted to the computer online in order to save memory. The computer is an i5-2320 CPU with 3.0 GHz and 32 G RAM. The MATLAB 2011 is used to run the method and share the data from the controller by data/file exchange technology. We add a white noise to data from the sensor before they are transmitted to the computer in order to strengthen the noise’s effects. The test bed of the DC-motor is shown in Figure 4.

A fault-free time series is produced according to the DC-motor system. The estimated model of one-order of system is obtained by LSM and has passed the statistical test under the significance level of 0.05 in the healthy condition:

(53) $$\left[\begin{array}{c} i_{a}\left(k+1\right)\\ \omega_{\beta }\left(k+1\right) \end{array}\right]=\left[\begin{array}{ccc} 0.4261 & 0.0030 & 0.0123\\ 1.3430 & 0.9910 & 0.0329 \end{array}\right]\left[\begin{array}{c} i_{a}\left(k\right)\\ \omega_{\beta }\left(k\right)\\ u(k) \end{array}\right]$$

### 4.1. Swift Detection

**Figure 5:**
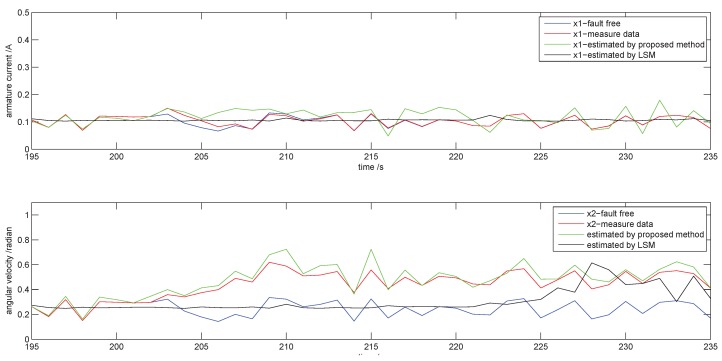
The evolution of states (from 195–235).

Firstly, we do an experiment to test the speediness of fault judgement. The fault signal $\omega_{f}$ with a step of amplitude 0.2 is added to State x2 from Sample 200. The results from Sample 195 to Sample 235 are shown in Figure 5. The blue curve, the red curve and the green curve are the data that are fault free, measured data subject to fault and estimated data by the proposed method, respectively. When the fault occurs, the system responds to the fault after two sampling periods due to the inertia. State x1 conforms to the healthy state (blue curve) due to the little influence of this fault. State x2 begins to deviate from the blue curve from Sample 203 and raises to 0.5 after seven sampling periods. The new stability that has a stable bias with the healthy state (blue curve) achieves at the time of system response the stability of the fault. The estimated data (green curve) for the RL method are obtained by immediately adjusting the model parameters along with minimizing the NSR. One can see that the green curve coincides with the red curve whether before and after a fault occurs.

In order to compare with the sliding window method (SLW), we determine an estimated $\hat{\theta }$ instead of θ by LSM with the width of sliding window l = 50. The result is shown in Figure 5 as the black curve. The black curve shows that State x2 has a similar tendency as the green curve except with a delay. During the healthy stage, both SLW and RL methods have good performance in tracing measure data (red curve), and the SLW has less fluctuations than RL. When a fault appears, the SLM will experience a transient process similar to the green curve, raising from 0.3–0.5 after about 25 sampling periods, but not immediately. This means the SLM will have a longer delay to respond to the fault. The SLW method is good for the healthy process that has a stable statistical indicator. When there is a fault occurrence, the statistical indicators of the data series move to a new stable state to fit the fault after they suffer a transition change. This process depends on the fault style and intensity. Therefore, the SLW method cannot avoid the delay due to its necessary data collection to change the statistical indicators in the range of its window length. It can speed the judgement by shortening the window length. However, if the window length is too small, the statistical indicators will become unstable because the data of the window cannot express the feature of the data series. Our proposed RL method will make up for this condition.

**Figure 6:**
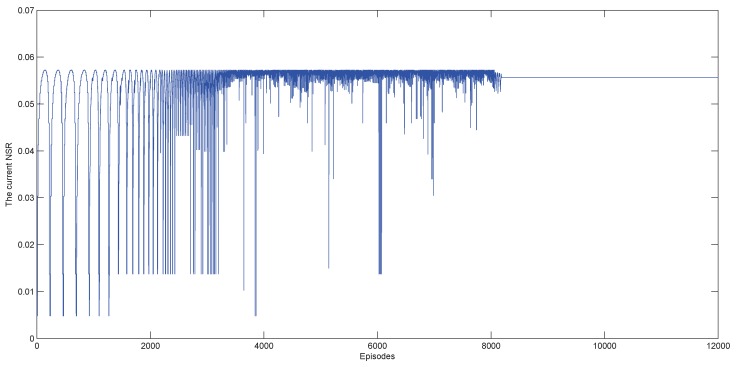
The training process.

We also show a training process of minimizing the noise-signal ratio by reinforcement learning. It is seen in Figure 6. The horizontal coordinate and vertical coordinate represent the episodes and the responding NSR, respectively. The discount factor γ is 0.95. Beginning with a parameter ${\hat{\theta }}_{f}\left(k\right)$ randomly (as Procedure 1), the NSR will converge after a training of 8300 episodes, and one will get the required parameter ${\hat{\theta }}_{f}\left(k\right)$ when it is convergent.

### 4.2. Fault Detection

**Figure 7:**
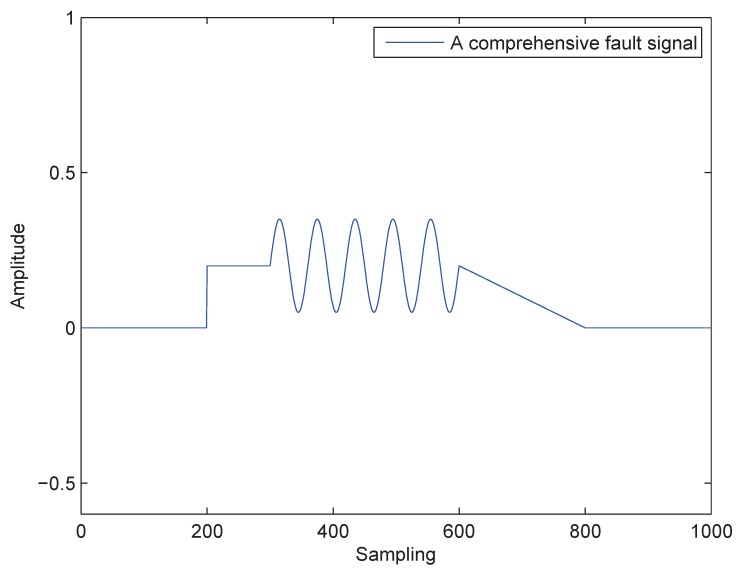
The fault signal.

A comprehensive fault signal $\omega_{f}$ combined with a step, a sine and a slope is added to State x2 in order to verify the fault diagnosis and detection ability of the proposed RL method. The fault signal is generated according to Equation (54):

(54) $$\omega_{f}\left(k\right)=\left\{\begin{array}{ll} 0 & 0<k\leq 200\\ 0.2 & 200<k\leq 300\\ 0.2+0.15\sin(\pi (k-300)/30) & 300<k\leq 600\\ -0.001k+0.8 & 600<k\leq 800\\ 0 & 800<k\leq 1000 \end{array}\right.$$

and shown in Figure 7.

**Figure 8:**
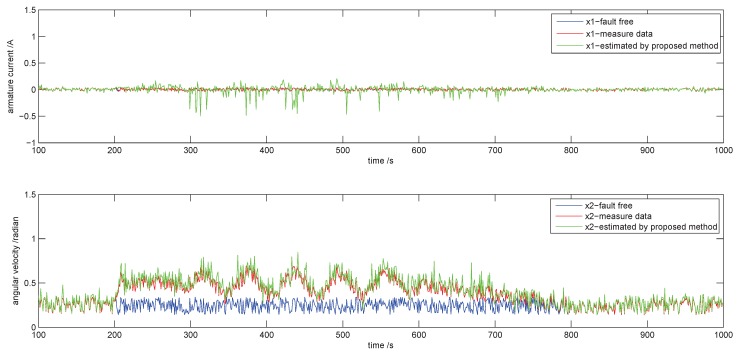
The evolution of states.

The state ${\hat{x}}_{f}\left(k+1\right)$ at time k+1 is estimated based on the observation $\phi_{f}\left(k\right)$ at time k according to Equation (15) and in which ${\hat{\theta }}_{f}$ is obtained by the proposed RL approach. The evolution of states from k=100 to k=1000 is shown in Figure 8. The blue curve, the red curve and the green curve are the data that are fault free, the measured data and the estimated data, respectively.

**Figure 9:**
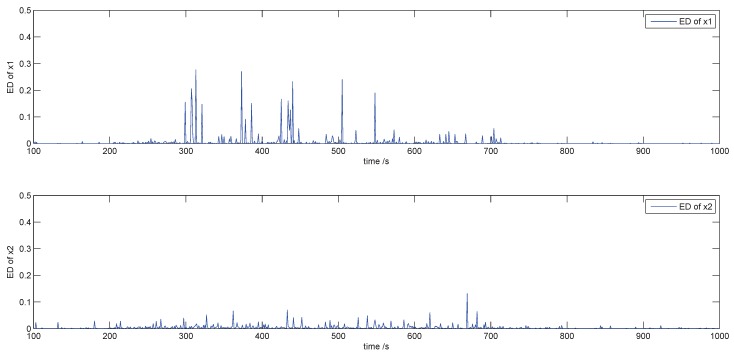
The error between measure and estimation.

It is seen from Figure 8 that the estimated data (green curve) coincide with the measure data (red curve) throughout the process of different faults. In fact, the green curve is an estimation based on the measured data at the previous moment by using the proposed RL approach. It is produced a sampling period earlier than the red curve. We also compute the errors between measurement and estimation according to Equation (49) in order to show the accuracy. The mean of x1 and x2 between measured data and estimated data are 0.05 and 0.02, respectively, and the maximum error is 0.25 and 0.15. The result is seen in Figure 9.

**Figure 10:**
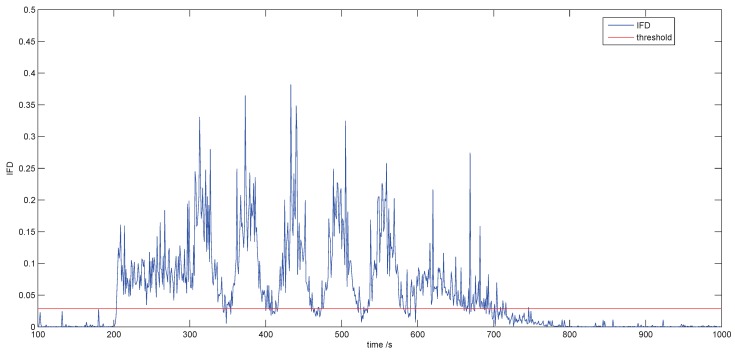
Results of fault detection. IFD, indicator of fault degree.

If the data that are fault free are taken as a reference and the fault degree is expressed with the IFD according to Equation (52), the threshold of ED is obtained in the condition of being fault free based on the healthy data from 1–200 by Equation (50) and $ED_{sh}=0.0286$. Then, we compute the IFDs at every sampling time according to Equation (52). The results are shown in Figure 10. The blue curve and the red curve are the indicator of fault degree (IFD) and the threshold of ED, respectively.

Figure 10 shows the IFD that is fault free is below the threshold. During the fault process, the IFD that fluctuates with a limited range is above the threshold except some samples that are close to healthy data.

We will also know the fault severity at every sample by observing the $IFD^{\prime }$s scale. For example, the fault from Sample 200–Sample 300 is limited between 0.05 and 0.15, which means the fault is comparatively stable. At Samples 320, 380, 440 and 510, a peak appears respectively with a heavy fault over 0.3.

### 4.3. Influence of Disturbance

**Figure 11:**
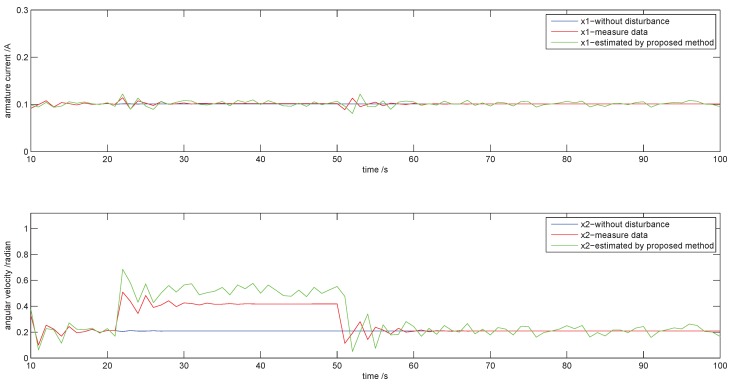
The evolution of states in disturbance.

We give a step disturbance to State x2 by raising the control voltage at Sample 20. The evolution of states is shown in Figure 11. The blue curve, the red curve and the green curve are data without disturbance, measured data and estimated data by the proposed method, respectively. It is seen that the armature current almost keeps the initial state because there is no load change. The angular velocity (red curve) rises to 0.4 rad in response to this disturbance after a short transition. The proposed method gives an ample estimation (green curve) because the data with disturbance have enlarged the NSR more that without disturbance in a long enough process. From an inverse view, an ample estimation will be taken to make up the NSR without disturbance according to the proposed method. This shows the RL’s robustness in disturbance.

The proposed method cannot distinguish between faults and disturbances because it makes a decision only according to the NSR. In fact, the disturbance is eliminated by the closed loop of the control system. If the disturbance cannot be removed by the control system due to the fault, it is necessary for this disturbance to be handled as a special fault in order to keep the plant safe and effective.

## 5. Conclusions

Comparing a single sample datum with healthy data is the fastest way for fault detection. However, it can hardly be achieved because the noise of sample data will disturb the normal data. No one knows whether the discrepancy between sample data and healthy data comes from fault or comes from noise only according to a single collected datum. The statistical method needs a quantity of valid data; however, it is difficult to obtain them in the early stage of unexpected fault, which leads to a dilemma of prompt FDD. In order to solve these shortages, a reinforcement learning method has been proposed to estimate the model parameter by taking the parameter as a special action. Taking a minimization of the NSR as a goal of the data series, the model parameter can be obtained by applying the technology of the policy valuation and policy improvement. This method has the ability of getting rid of the noise’s influence and keeping consistency with the current situation. Furthermore, the FDD has been implemented by evaluating the residual of the real-time process data and pre-obtained healthy time-series data. The fault can be promptly detected with the help of the threshold from the healthy data series by only using the information within one sampling period.

In the future, further work will distinguish the slight fault signal from healthy data as quickly as possible and apply this method to an engineering-oriented real-time process.
